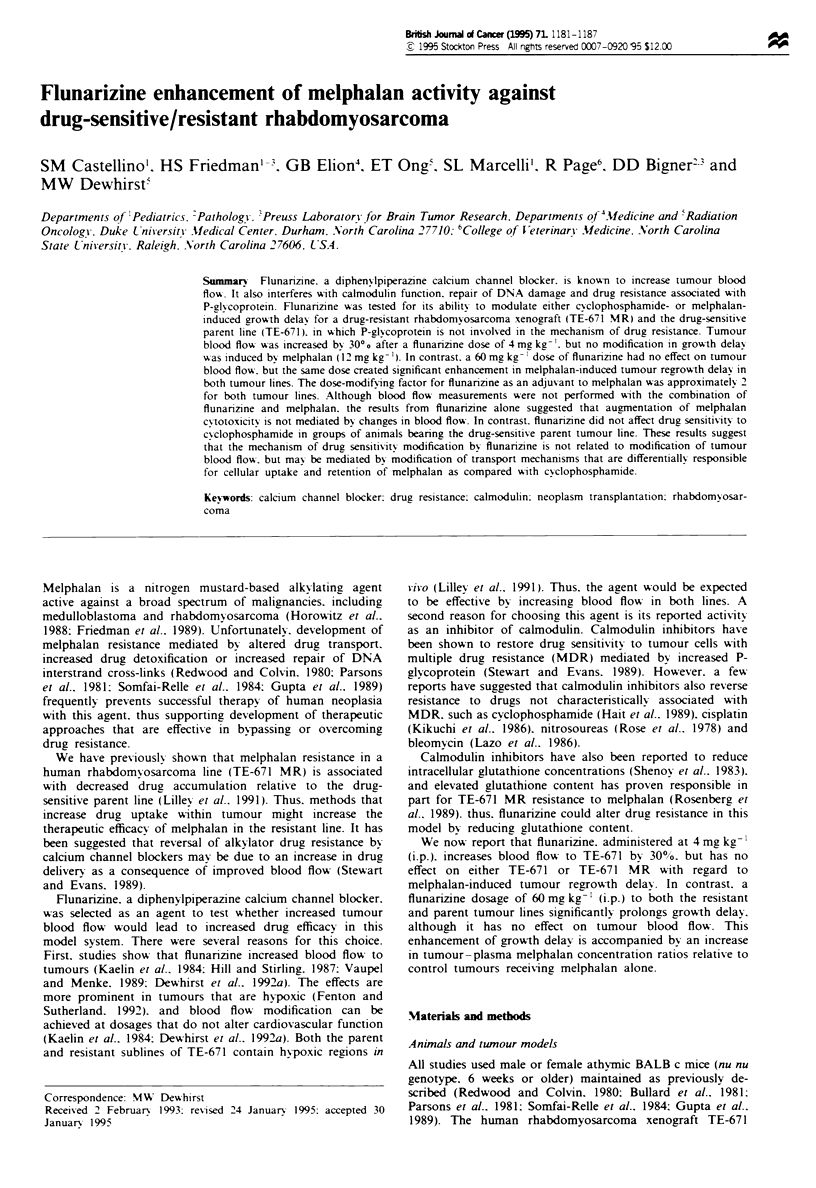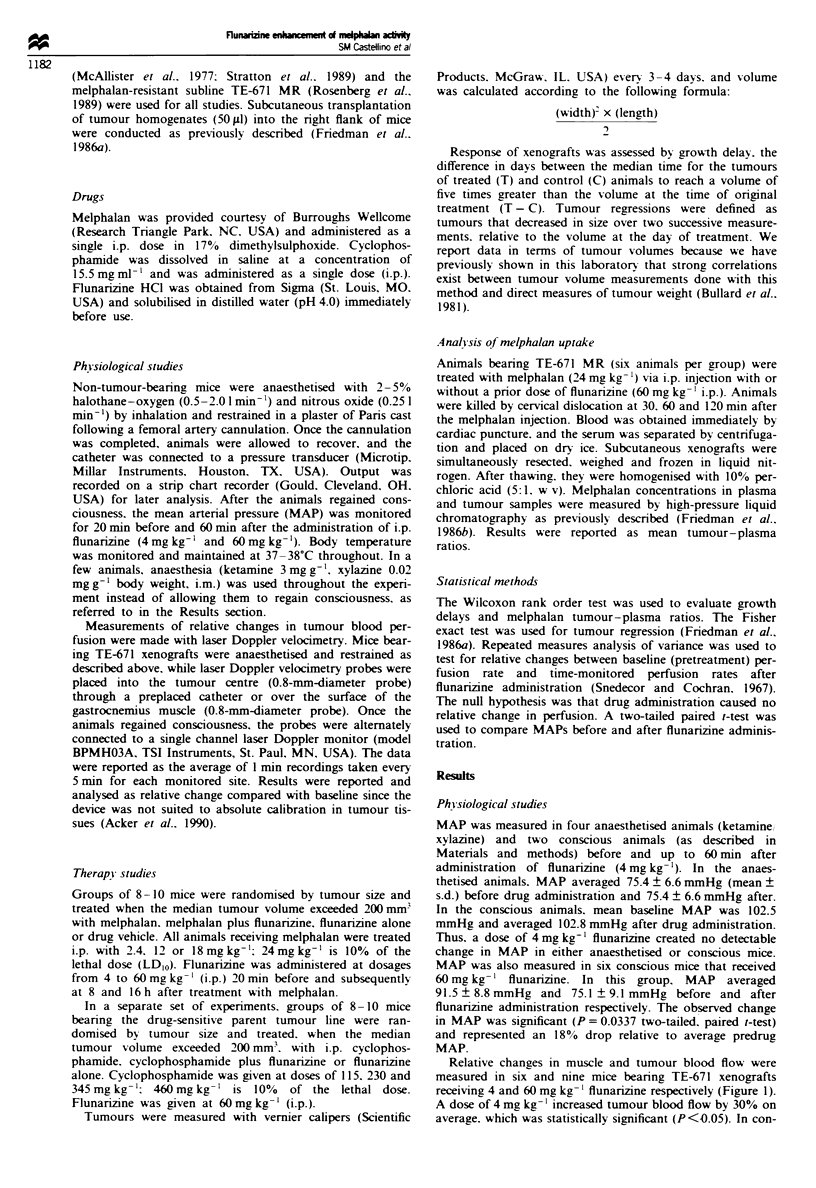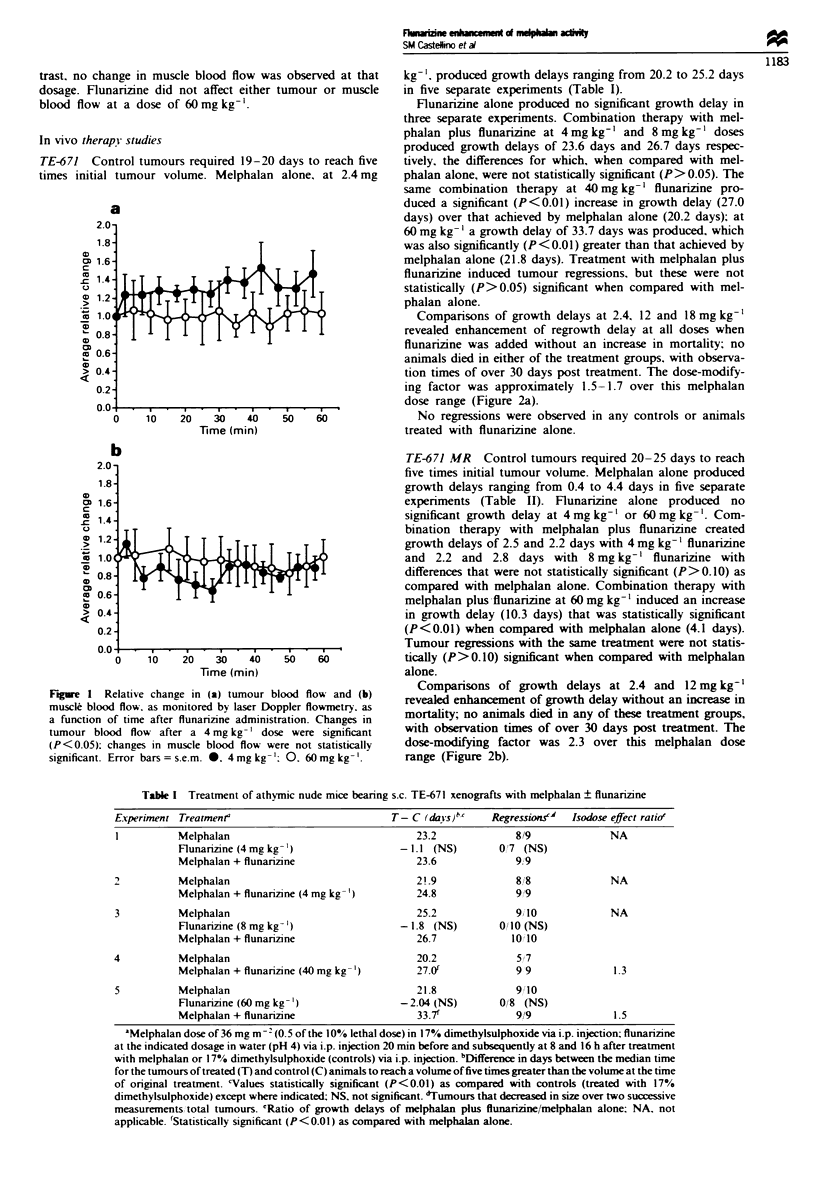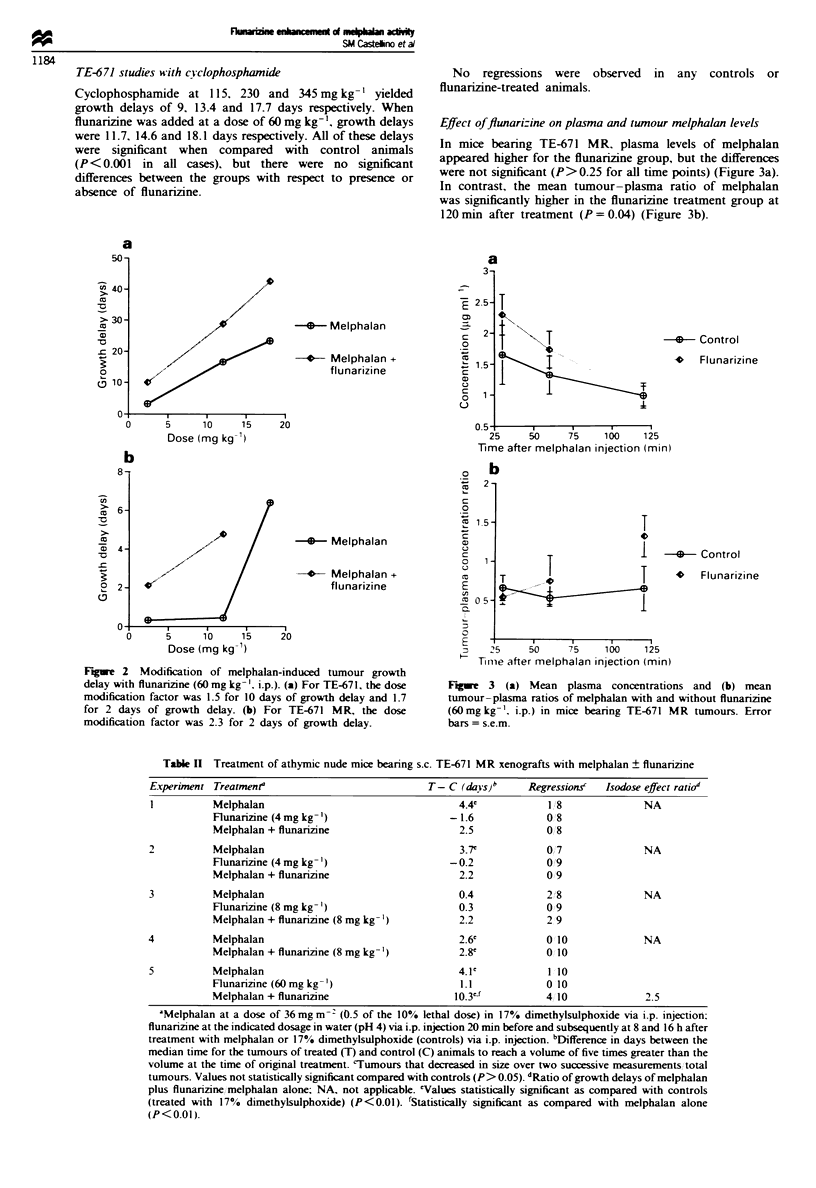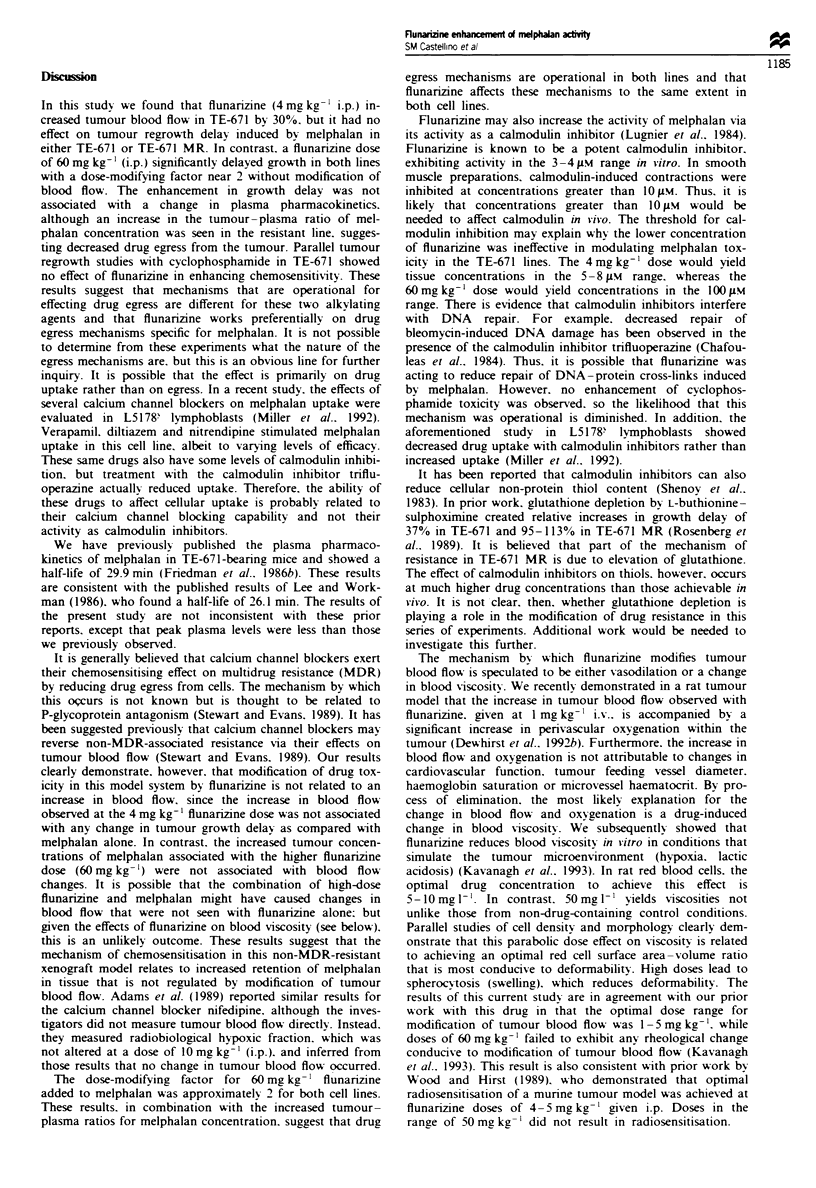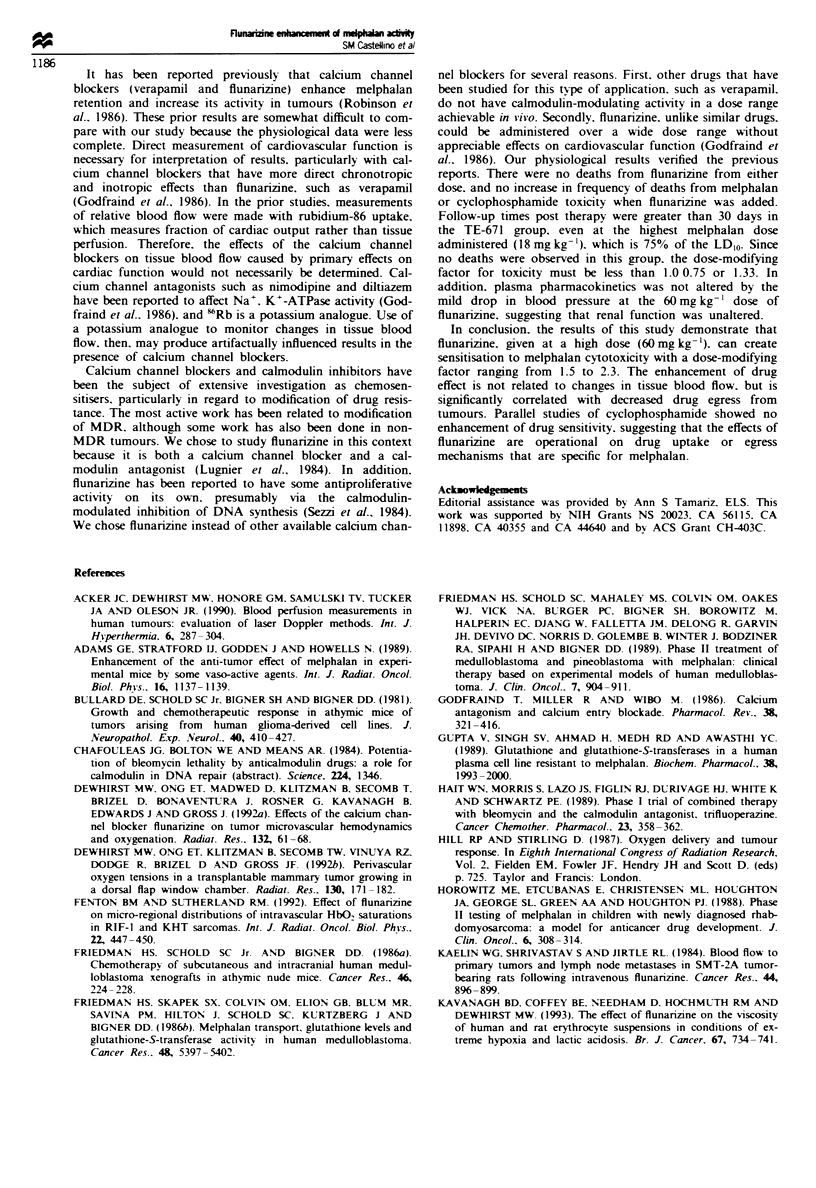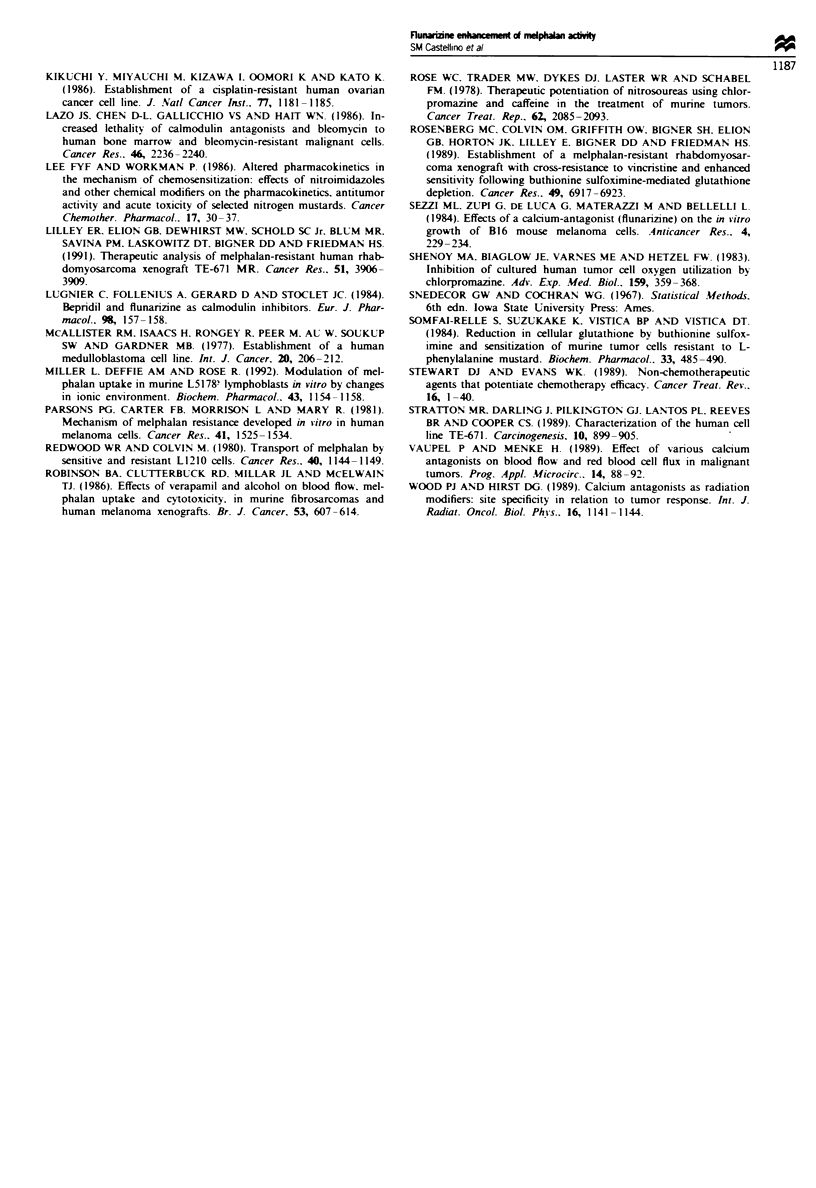# Flunarizine enhancement of melphalan activity against drug-sensitive/resistant rhabdomyosarcoma.

**DOI:** 10.1038/bjc.1995.230

**Published:** 1995-06

**Authors:** S. M. Castellino, H. S. Friedman, G. B. Elion, E. T. Ong, S. L. Marcelli, R. Page, D. D. Bigner, M. W. Dewhirst

**Affiliations:** Department of Pediatrics, Duke University Medical Center, Durham, North Carolina 27710, USA.

## Abstract

Flunarizine, a diphenylpiperazine calcium channel blocker, is known to increase tumor blood flow. It also interferes with calmodulin function, repair of DNA damage and drug resistance associated with P-glycoprotein. Flunarizine was tested for its ability to modulate either cyclophosphamide- or melphalan-induced growth delay for a drug-resistant rhabdomyosarcoma xenograft (TE-671 MR) and the drug-sensitive parent line (TE-671), in which P-glycoprotein is not involved in the mechanism of drug resistance. Tumour blood flow was increased by 30% after a flunarizine dose of 4 mg kg-1, but no modification in growth delay was induced by melphalan (12 mg kg-1). In contrast, a 60 mg kg-1 dose of flunarizine had no effect on tumour blood flow, but the same dose created significant enhancement in melphalan-induced tumour regrowth delay in both tumour lines. The dose-modifying factor for flunarizine as an adjuvant to melphalan was approximately 2 for both tumour lines. Although blood flow measurements were not performed with the combination of flunarizine and melphalan, the results from flunarizine alone suggested that augmentation of melphalan cytotoxicity is not mediated by changes in blood flow. In contrast, flunarizine did not affect drug sensitivity to cyclophosphamide in groups of animals bearing the drug-sensitive parent tumour line. These results suggest that the mechanism of drug sensitivity modification by flunarizine is not related to modification of tumour blood flow, but may be mediated by modification of transport mechanisms that are differentially responsible for cellular uptake and retention of melphalan as compared with cyclophosphamide.


British Journal of Cancer (1995) 71, 1181-1187

© 1995 Stockton Press All rights reserved 0007-0920/95 $12.00

### Flunarizine enhancement of melphalan activity against drug-sensitive/resistant rhabdomyosarcoma

SM Castellino[1], HS Friedman[1-3], GB Elion[4], ET Ong[5], SL Marcelli[1], R Page[6], DD Bigner[2,3] and MW Dewhirst[5]

*Departments of [1]Pediatrics, [2]Pathology, [3]Preuss Laboratory for Brain Tumor Research, Departments of [4]Medicine and [5]Radiation Oncology, Duke University Medical Center, Durham, North Carolina 27710; [6]College of Veterinary Medicine, North Carolina State University, Raleigh, North Carolina 27606, USA.*

**Summary** Flunarizine, a diphenylpiperazine calcium channel blocker, is known to increase tumour blood flow. It also interferes with calmodulin function, repair of DNA damage and drug resistance associated with P-glycoprotein. Flunarizine was tested for its ability to modulate either cyclophosphamide- or melphalan-induced growth delay for a drug-resistant rhabdomyosarcoma xenograft (TE-671 MR) and the drug-sensitive parent line (TE-671), in which P-glycoprotein is not involved in the mechanism of drug resistance. Tumour blood flow was increased by 30% after a flunarizine dose of 4 mg kg$^{-1}$, but no modification in growth delay was induced by melphalan (12 mg kg$^{-1}$). In contrast, a 60 mg kg$^{-1}$ dose of flunarizine had no effect on tumour blood flow, but the same dose created significant enhancement in melphalan-induced tumour regrowth delay in both tumour lines. The dose-modifying factor for flunarizine as an adjuvant to melphalan was approximately 2 for both tumour lines. Although blood flow measurements were not performed with the combination of flunarizine and melphalan, the results from flunarizine alone suggested that augmentation of melphalan cytotoxicity is not mediated by changes in blood flow. In contrast, flunarizine did not affect drug sensitivity to cyclophosphamide in groups of animals bearing the drug-sensitive parent tumour line. These results suggest that the mechanism of drug sensitivity modification by flunarizine is not related to modification of tumour blood flow, but may be mediated by modification of transport mechanisms that are differentially responsible for cellular uptake and retention of melphalan as compared with cyclophosphamide.

**Keywords**: calcium channel blocker; drug resistance; calmodulin; neoplasm transplantation; rhabdomyosarcoma

Melphalan is a nitrogen mustard-based alkylating agent active against a broad spectrum of malignancies, including medulloblastoma and rhabdomyosarcoma (Horowitz *et al.*, 1988; Friedman *et al.*, 1989). Unfortunately, development of melphalan resistance mediated by altered drug transport, increased drug detoxification or increased repair of DNA interstrand cross-links (Redwood and Colvin, 1980; Parsons *et al.*, 1981; Somfai-Relle *et al.*, 1984; Gupta *et al.*, 1989) frequently prevents successful therapy of human neoplasia with this agent, thus supporting development of therapeutic approaches that are effective in bypassing or overcoming drug resistance.

We have previously shown that melphalan resistance in a human rhabdomyosarcoma line (TE-671 MR) is associated with decreased drug accumulation relative to the drug-sensitive parent line (Lilley *et al.*, 1991). Thus, methods that increase drug uptake within tumour might increase the therapeutic efficacy of melphalan in the resistant line. It has been suggested that reversal of alkylator drug resistance by calcium channel blockers may be due to an increase in drug delivery as a consequence of improved blood flow (Stewart and Evans, 1989).

Flunarizine, a diphenylpiperazine calcium channel blocker, was selected as an agent to test whether increased tumour blood flow would lead to increased drug efficacy in this model system. There were several reasons for this choice. First, studies show that flunarizine increased blood flow to tumours (Kaelin *et al.*, 1984; Hill and Stirling, 1987; Vaupel and Menke, 1989; Dewhirst *et al.*, 1992*a*). The effects are more prominent in tumours that are hypoxic (Fenton and Sutherland, 1992), and blood flow modification can be achieved at dosages that do not alter cardiovascular function (Kaelin *et al.*, 1984; Dewhirst *et al.*, 1992*a*). Both the parent and resistant sublines of TE-671 contain hypoxic regions *in vivo* (Lilley *et al.*, 1991). Thus, the agent would be expected to be effective by increasing blood flow in both lines. A second reason for choosing this agent is its reported activity as an inhibitor of calmodulin. Calmodulin inhibitors have been shown to restore drug sensitivity to tumour cells with multiple drug resistance (MDR) mediated by increased P-glycoprotein (Stewart and Evans, 1989). However, a few reports have suggested that calmodulin inhibitors also reverse resistance to drugs not characteristically associated with MDR, such as cyclophosphamide (Hait *et al.*, 1989), cisplatin (Kikuchi *et al.*, 1986), nitrosoureas (Rose *et al.*, 1978) and bleomycin (Lazo *et al.*, 1986).

Calmodulin inhibitors have also been reported to reduce intracellular glutathione concentrations (Shenoy *et al.*, 1983), and elevated glutathione content has proven responsible in part for TE-671 MR resistance to melphalan (Rosenberg *et al.*, 1989), thus, flunarizine could alter drug resistance in this model by reducing glutathione content.

We now report that flunarizine, administered at 4 mg kg$^{-1}$ (i.p.), increases blood flow to TE-671 by 30%, but has no effect on either TE-671 or TE-671 MR with regard to melphalan-induced tumour regrowth delay. In contrast, a flunarizine dosage of 60 mg kg$^{-1}$ (i.p.) to both the resistant and parent tumour lines significantly prolongs growth delay, although it has no effect on tumour blood flow. This enhancement of growth delay is accompanied by an increase in tumour-plasma melphalan concentration ratios relative to control tumours receiving melphalan alone.

### Materials and methods

### Animals and tumour models

All studies used male or female athymic BALB c mice (*nu nu* genotype, 6 weeks or older) maintained as previously described (Redwood and Colvin, 1980; Bullard *et al.*, 1981; Parsons *et al.*, 1981; Somfai-Relle *et al.*, 1984; Gupta *et al.*, 1989). The human rhabdomyosarcoma xenograft TE-671

Correspondence: MW Dewhirst
Received 2 February 1993; revised 24 January 1995; accepted 30 January 1995

(McAllister et al., 1977; Stratton et al., 1989) and the melphalan-resistant subline TE-671 MR (Rosenberg et al., 1989) were used for all studies. Subcutaneous transplantation of tumour homogenates (50 μl) into the right flank of mice were conducted as previously described (Friedman et al., 1986a).

*Drugs*

Melphalan was provided courtesy of Burroughs Wellcome (Research Triangle Park, NC, USA) and administered as a single i.p. dose in 17% dimethylsulphoxide. Cyclophosphamide was dissolved in saline at a concentration of 15.5 mg ml$^{-1}$ and was administered as a single dose (i.p.). Flunarizine HCl was obtained from Sigma (St. Louis, MO, USA) and solubilised in distilled water (pH 4.0) immediately before use.

*Physiological studies*

Non-tumour-bearing mice were anaesthetised with 2–5% halothane–oxygen (0.5–2.0 l min$^{-1}$) and nitrous oxide (0.25 l min$^{-1}$) by inhalation and restrained in a plaster of Paris cast following a femoral artery cannulation. Once the cannulation was completed, animals were allowed to recover, and the catheter was connected to a pressure transducer (Microtip, Millar Instruments, Houston, TX, USA). Output was recorded on a strip chart recorder (Gould, Cleveland, OH, USA) for later analysis. After the animals regained consciousness, the mean arterial pressure (MAP) was monitored for 20 min before and 60 min after the administration of i.p. flunarizine (4 mg kg$^{-1}$ and 60 mg kg$^{-1}$). Body temperature was monitored and maintained at 37–38°C throughout. In a few animals, anaesthesia (ketamine 3 mg g$^{-1}$, xylazine 0.02 mg g$^{-1}$ body weight, i.m.) was used throughout the experiment instead of allowing them to regain consciousness, as referred to in the Results section.

Measurements of relative changes in tumour blood perfusion were made with laser Doppler velocimetry. Mice bearing TE-671 xenografts were anaesthetised and restrained as described above, while laser Doppler velocimetry probes were placed into the tumour centre (0.8-mm-diameter probe) through a preplaced catheter or over the surface of the gastrocnemius muscle (0.8-mm-diameter probe). Once the animals regained consciousness, the probes were alternately connected to a single channel laser Doppler monitor (model BPMH03A, TSI Instruments, St. Paul, MN, USA). The data were reported as the average of 1 min recordings taken every 5 min for each monitored site. Results were reported and analysed as relative change compared with baseline since the device was not suited to absolute calibration in tumour tissues (Acker et al., 1990).

*Therapy studies*

Groups of 8–10 mice were randomised by tumour size and treated when the median tumour volume exceeded 200 mm$^3$ with melphalan, melphalan plus flunarizine, flunarizine alone or drug vehicle. All animals receiving melphalan were treated i.p. with 2.4, 12 or 18 mg kg$^{-1}$; 24 mg kg$^{-1}$ is 10% of the lethal dose ($LD_{10}$). Flunarizine was administered at dosages from 4 to 60 mg kg$^{-1}$ (i.p.) 20 min before and subsequently at 8 and 16 h after treatment with melphalan.

In a separate set of experiments, groups of 8–10 mice bearing the drug-sensitive parent tumour line were randomised by tumour size and treated, when the median tumour volume exceeded 200 mm$^3$, with i.p. cyclophosphamide, cyclophosphamide plus flunarizine or flunarizine alone. Cyclophosphamide was given at doses of 115, 230 and 345 mg kg$^{-1}$; 460 mg kg$^{-1}$ is 10% of the lethal dose. Flunarizine was given at 60 mg kg$^{-1}$ (i.p.).

Tumours were measured with vernier calipers (Scientific Products, McGraw, IL, USA) every 3–4 days, and volume was calculated according to the following formula:

$$\frac{(\text{width})^2 \times (\text{length})}{2}$$

Response of xenografts was assessed by growth delay, the difference in days between the median time for the tumours of treated (T) and control (C) animals to reach a volume of five times greater than the volume at the time of original treatment (T – C). Tumour regressions were defined as tumours that decreased in size over two successive measurements, relative to the volume at the day of treatment. We report data in terms of tumour volumes because we have previously shown in this laboratory that strong correlations exist between tumour volume measurements done with this method and direct measures of tumour weight (Bullard et al., 1981).

*Analysis of melphalan uptake*

Animals bearing TE-671 MR (six animals per group) were treated with melphalan (24 mg kg$^{-1}$) via i.p. injection with or without a prior dose of flunarizine (60 mg kg$^{-1}$ i.p.). Animals were killed by cervical dislocation at 30, 60 and 120 min after the melphalan injection. Blood was obtained immediately by cardiac puncture, and the serum was separated by centrifugation and placed on dry ice. Subcutaneous xenografts were simultaneously resected, weighed and frozen in liquid nitrogen. After thawing, they were homogenised with 10% perchloric acid (5:1, w/v). Melphalan concentrations in plasma and tumour samples were measured by high-pressure liquid chromatography as previously described (Friedman et al., 1986b). Results were reported as mean tumour–plasma ratios.

*Statistical methods*

The Wilcoxon rank order test was used to evaluate growth delays and melphalan tumour–plasma ratios. The Fisher exact test was used for tumour regression (Friedman et al., 1986a). Repeated measures analysis of variance was used to test for relative changes between baseline (pretreatment) perfusion rate and time-monitored perfusion rates after flunarizine administration (Snedecor and Cochran, 1967). The null hypothesis was that drug administration caused no relative change in perfusion. A two-tailed paired t-test was used to compare MAPs before and after flunarizine administration.

### Results

*Physiological studies*

MAP was measured in four anaesthetised animals (ketamine/xylazine) and two conscious animals (as described in Materials and methods) before and up to 60 min after administration of flunarizine (4 mg kg$^{-1}$). In the anaesthetised animals, MAP averaged 75.4 ± 6.6 mmHg (mean ± s.d.) before drug administration and 75.4 ± 6.6 mmHg after. In the conscious animals, mean baseline MAP was 102.5 mmHg and averaged 102.8 mmHg after drug administration. Thus, a dose of 4 mg kg$^{-1}$ flunarizine created no detectable change in MAP in either anaesthetised or conscious mice. MAP was also measured in six conscious mice that received 60 mg kg$^{-1}$ flunarizine. In this group, MAP averaged 91.5 ± 8.8 mmHg and 75.1 ± 9.1 mmHg before and after flunarizine administration respectively. The observed change in MAP was significant ($P = 0.0337$ two-tailed, paired t-test) and represented an 18% drop relative to average predrug MAP.

Relative changes in muscle and tumour blood flow were measured in six and nine mice bearing TE-671 xenografts receiving 4 and 60 mg kg$^{-1}$ flunarizine respectively (Figure 1). A dose of 4 mg kg$^{-1}$ increased tumour blood flow by 30% on average, which was statistically significant ($P < 0.05$). In contrast, no change in muscle blood flow was observed at that dosage. Flunarizine did not affect either tumour or muscle blood flow at a dose of 60 mg kg$^{-1}$.

### In vivo *therapy studies*

*TE-671* Control tumours required 19–20 days to reach five times initial tumour volume. Melphalan alone, at 2.4 mg

**Figure 1** Relative change in (**a**) tumour blood flow and (**b**) muscle blood flow, as monitored by laser Doppler flowmetry, as a function of time after flunarizine administration. Changes in tumour blood flow after a 4 mg kg$^{-1}$ dose were significant ($P<0.05$); changes in muscle blood flow were not statistically significant. Error bars = s.e.m. ●, 4 mg kg$^{-1}$; ○, 60 mg kg$^{-1}$.

kg$^{-1}$, produced growth delays ranging from 20.2 to 25.2 days in five separate experiments (Table I).

Flunarizine alone produced no significant growth delay in three separate experiments. Combination therapy with melphalan plus flunarizine at 4 mg kg$^{-1}$ and 8 mg kg$^{-1}$ doses produced growth delays of 23.6 days and 26.7 days respectively, the differences for which, when compared with melphalan alone, were not statistically significant ($P>0.05$). The same combination therapy at 40 mg kg$^{-1}$ flunarizine produced a significant ($P<0.01$) increase in growth delay (27.0 days) over that achieved by melphalan alone (20.2 days); at 60 mg kg$^{-1}$ a growth delay of 33.7 days was produced, which was also significantly ($P<0.01$) greater than that achieved by melphalan alone (21.8 days). Treatment with melphalan plus flunarizine induced tumour regressions, but these were not statistically ($P>0.05$) significant when compared with melphalan alone.

Comparisons of growth delays at 2.4, 12 and 18 mg kg$^{-1}$ revealed enhancement of regrowth delay at all doses when flunarizine was added without an increase in mortality; no animals died in either of the treatment groups, with observation times of over 30 days post treatment. The dose-modifying factor was approximately 1.5–1.7 over this melphalan dose range (Figure 2a).

No regressions were observed in any controls or animals treated with flunarizine alone.

*TE-671 MR* Control tumours required 20–25 days to reach five times initial tumour volume. Melphalan alone produced growth delays ranging from 0.4 to 4.4 days in five separate experiments (Table II). Flunarizine alone produced no significant growth delay at 4 mg kg$^{-1}$ or 60 mg kg$^{-1}$. Combination therapy with melphalan plus flunarizine created growth delays of 2.5 and 2.2 days with 4 mg kg$^{-1}$ flunarizine and 2.2 and 2.8 days with 8 mg kg$^{-1}$ flunarizine with differences that were not statistically significant ($P>0.10$) as compared with melphalan alone. Combination therapy with melphalan plus flunarizine at 60 mg kg$^{-1}$ induced an increase in growth delay (10.3 days) that was statistically significant ($P<0.01$) when compared with melphalan alone (4.1 days). Tumour regressions with the same treatment were not statistically ($P>0.10$) significant when compared with melphalan alone.

Comparisons of growth delays at 2.4 and 12 mg kg$^{-1}$ revealed enhancement of growth delay without an increase in mortality; no animals died in any of these treatment groups, with observation times of over 30 days post treatment. The dose-modifying factor was 2.3 over this melphalan dose range (Figure 2b).

**Table I** Treatment of athymic nude mice bearing s.c. TE-671 xenografts with melphalan ± flunarizine

| *Experiment* | *Treatment*[a] | *T − C (days)*[b,c] | *Regressions*[c,d] | *Isodose effect ratio*[e] |
|---|---|---|---|---|
| 1 | Melphalan | 23.2 | 8/9 | NA |
| | Flunarizine (4 mg kg$^{-1}$) | −1.1 (NS) | 0/7 (NS) | |
| | Melphalan + flunarizine | 23.6 | 9/9 | |
| 2 | Melphalan | 21.9 | 8/8 | NA |
| | Melphalan + flunarizine (4 mg kg$^{-1}$) | 24.8 | 9/9 | |
| 3 | Melphalan | 25.2 | 9/10 | NA |
| | Flunarizine (8 mg kg$^{-1}$) | −1.8 (NS) | 0/10 (NS) | |
| | Melphalan + flunarizine | 26.7 | 10/10 | |
| 4 | Melphalan | 20.2 | 5/7 | |
| | Melphalan + flunarizine (40 mg kg$^{-1}$) | 27.0[f] | 9/9 | 1.3 |
| 5 | Melphalan | 21.8 | 9/10 | |
| | Flunarizine (60 mg kg$^{-1}$) | −2.04 (NS) | 0/8 (NS) | |
| | Melphalan + flunarizine | 33.7[f] | 9/9 | 1.5 |

[a]Melphalan dose of 36 mg m$^{-2}$ (0.5 of the 10% lethal dose) in 17% dimethylsulphoxide via i.p. injection; flunarizine at the indicated dosage in water (pH 4) via i.p. injection 20 min before and subsequently at 8 and 16 h after treatment with melphalan or 17% dimethylsulphoxide (controls) via i.p. injection. [b]Difference in days between the median time for the tumours of treated (T) and control (C) animals to reach a volume of five times greater than the volume at the time of original treatment. [c]Values statistically significant ($P<0.01$) as compared with controls (treated with 17% dimethylsulphoxide) except where indicated; NS, not significant. [d]Tumours that decreased in size over two successive measurements/total tumours. [e]Ratio of growth delays of melphalan plus flunarizine/melphalan alone; NA, not applicable. [f]Statistically significant ($P<0.01$) as compared with melphalan alone.

*TE-671 studies with cyclophosphamide*

Cyclophosphamide at 115, 230 and 345 mg $kg^{-1}$ yielded growth delays of 9, 13.4 and 17.7 days respectively. When flunarizine was added at a dose of 60 mg $kg^{-1}$, growth delays were 11.7, 14.6 and 18.1 days respectively. All of these delays were significant when compared with control animals ($P<0.001$ in all cases), but there were no significant differences between the groups with respect to presence or absence of flunarizine.

No regressions were observed in any controls or flunarizine-treated animals.

*Effect of flunarizine on plasma and tumour melphalan levels*

In mice bearing TE-671 MR, plasma levels of melphalan appeared higher for the flunarizine group, but the differences were not significant ($P>0.25$ for all time points) (Figure 3a). In contrast, the mean tumour-plasma ratio of melphalan was significantly higher in the flunarizine treatment group at 120 min after treatment ($P=0.04$) (Figure 3b).

**Figure 2** Modification of melphalan-induced tumour growth delay with flunarizine (60 mg $kg^{-1}$, i.p.). (**a**) For TE-671, the dose modification factor was 1.5 for 10 days of growth delay and 1.7 for 2 days of growth delay. (**b**) For TE-671 MR, the dose modification factor was 2.3 for 2 days of growth delay.

**Figure 3** (**a**) Mean plasma concentrations and (**b**) mean tumour-plasma ratios of melphalan with and without flunarizine (60 mg $kg^{-1}$, i.p.) in mice bearing TE-671 MR tumours. Error bars = s.e.m.

**Table II** Treatment of athymic nude mice bearing s.c. TE-671 MR xenografts with melphalan ± flunarizine

| *Experiment* | *Treatment*[a] | *T − C (days)*[b] | *Regressions*[c] | *Isodose effect ratio*[d] |
|---|---|---|---|---|
| 1 | Melphalan | 4.4[e] | 1/8 | NA |
| | Flunarizine (4 mg $kg^{-1}$) | −1.6 | 0/8 | |
| | Melphalan + flunarizine | 2.5 | 0/8 | |
| 2 | Melphalan | 3.7[e] | 0/7 | NA |
| | Flunarizine (4 mg $kg^{-1}$) | −0.2 | 0/9 | |
| | Melphalan + flunarizine | 2.2 | 0/9 | |
| 3 | Melphalan | 0.4 | 2/8 | NA |
| | Flunarizine (8 mg $kg^{-1}$) | 0.3 | 0/9 | |
| | Melphalan + flunarizine (8 mg $kg^{-1}$) | 2.2 | 2/9 | |
| 4 | Melphalan | 2.6[e] | 0/10 | NA |
| | Melphalan + flunarizine (8 mg $kg^{-1}$) | 2.8[e] | 0/10 | |
| 5 | Melphalan | 4.1[e] | 1/10 | |
| | Flunarizine (60 mg $kg^{-1}$) | 1.1 | 0/10 | |
| | Melphalan + flunarizine | 10.3[e,f] | 4/10 | 2.5 |

[a]Melphalan at a dose of 36 mg $m^{-2}$ (0.5 of the 10% lethal dose) in 17% dimethylsulphoxide via i.p. injection; flunarizine at the indicated dosage in water (pH 4) via i.p. injection 20 min before and subsequently at 8 and 16 h after treatment with melphalan or 17% dimethylsulphoxide (controls) via i.p. injection. [b]Difference in days between the median time for the tumours of treated (T) and control (C) animals to reach a volume of five times greater than the volume at the time of original treatment. [c]Tumours that decreased in size over two successive measurements/total tumours. Values not statistically significant compared with controls ($P>0.05$). [d]Ratio of growth delays of melphalan plus flunarizine/melphalan alone; NA, not applicable. [e]Values statistically significant as compared with controls (treated with 17% dimethylsulphoxide) ($P<0.01$). [f]Statistically significant as compared with melphalan alone ($P<0.01$).

### Discussion

In this study we found that flunarizine ($4\,mg\,kg^{-1}$ i.p.) increased tumour blood flow in TE-671 by 30%, but it had no effect on tumour regrowth delay induced by melphalan in either TE-671 or TE-671 MR. In contrast, a flunarizine dose of $60\,mg\,kg^{-1}$ (i.p.) significantly delayed growth in both lines with a dose-modifying factor near 2 without modification of blood flow. The enhancement in growth delay was not associated with a change in plasma pharmacokinetics, although an increase in the tumour–plasma ratio of melphalan concentration was seen in the resistant line, suggesting decreased drug egress from the tumour. Parallel tumour regrowth studies with cyclophosphamide in TE-671 showed no effect of flunarizine in enhancing chemosensitivity. These results suggest that mechanisms that are operational for effecting drug egress are different for these two alkylating agents and that flunarizine works preferentially on drug egress mechanisms specific for melphalan. It is not possible to determine from these experiments what the nature of the egress mechanisms are, but this is an obvious line for further inquiry. It is possible that the effect is primarily on drug uptake rather than on egress. In a recent study, the effects of several calcium channel blockers on melphalan uptake were evaluated in L5178Y lymphoblasts (Miller *et al*., 1992). Verapamil, diltiazem and nitrendipine stimulated melphalan uptake in this cell line, albeit to varying levels of efficacy. These same drugs also have some levels of calmodulin inhibition, but treatment with the calmodulin inhibitor trifluoperazine actually reduced uptake. Therefore, the ability of these drugs to affect cellular uptake is probably related to their calcium channel blocking capability and not their activity as calmodulin inhibitors.

We have previously published the plasma pharmacokinetics of melphalan in TE-671-bearing mice and showed a half-life of 29.9 min (Friedman *et al*., 1986*b*). These results are consistent with the published results of Lee and Workman (1986), who found a half-life of 26.1 min. The results of the present study are not inconsistent with these prior reports, except that peak plasma levels were less than those we previously observed.

It is generally believed that calcium channel blockers exert their chemosensitising effect on multidrug resistance (MDR) by reducing drug egress from cells. The mechanism by which this occurs is not known but is thought to be related to P-glycoprotein antagonism (Stewart and Evans, 1989). It has been suggested previously that calcium channel blockers may reverse non-MDR-associated resistance via their effects on tumour blood flow (Stewart and Evans, 1989). Our results clearly demonstrate, however, that modification of drug toxicity in this model system by flunarizine is not related to an increase in blood flow, since the increase in blood flow observed at the $4\,mg\,kg^{-1}$ flunarizine dose was not associated with any change in tumour growth delay as compared with melphalan alone. In contrast, the increased tumour concentrations of melphalan associated with the higher flunarizine dose ($60\,mg\,kg^{-1}$) were not associated with blood flow changes. It is possible that the combination of high-dose flunarizine and melphalan might have caused changes in blood flow that were not seen with flunarizine alone; but given the effects of flunarizine on blood viscosity (see below), this is an unlikely outcome. These results suggest that the mechanism of chemosensitisation in this non-MDR-resistant xenograft model relates to increased retention of melphalan in tissue that is not regulated by modification of tumour blood flow. Adams *et al*. (1989) reported similar results for the calcium channel blocker nifedipine, although the investigators did not measure tumour blood flow directly. Instead, they measured radiobiological hypoxic fraction, which was not altered at a dose of $10\,mg\,kg^{-1}$ (i.p.), and inferred from those results that no change in tumour blood flow occurred.

The dose-modifying factor for $60\,mg\,kg^{-1}$ flunarizine added to melphalan was approximately 2 for both cell lines. These results, in combination with the increased tumour–plasma ratios for melphalan concentration, suggest that drug egress mechanisms are operational in both lines and that flunarizine affects these mechanisms to the same extent in both cell lines.

Flunarizine may also increase the activity of melphalan via its activity as a calmodulin inhibitor (Lugnier *et al*., 1984). Flunarizine is known to be a potent calmodulin inhibitor, exhibiting activity in the 3–4 $\mu$M range *in vitro*. In smooth muscle preparations, calmodulin-induced contractions were inhibited at concentrations greater than 10 $\mu$M. Thus, it is likely that concentrations greater than 10 $\mu$M would be needed to affect calmodulin *in vivo*. The threshold for calmodulin inhibition may explain why the lower concentration of flunarizine was ineffective in modulating melphalan toxicity in the TE-671 lines. The $4\,mg\,kg^{-1}$ dose would yield tissue concentrations in the 5–8 $\mu$M range, whereas the $60\,mg\,kg^{-1}$ dose would yield concentrations in the 100 $\mu$M range. There is evidence that calmodulin inhibitors interfere with DNA repair. For example, decreased repair of bleomycin-induced DNA damage has been observed in the presence of the calmodulin inhibitor trifluoperazine (Chafouleas *et al*., 1984). Thus, it is possible that flunarizine was acting to reduce repair of DNA–protein cross-links induced by melphalan. However, no enhancement of cyclophosphamide toxicity was observed, so the likelihood that this mechanism was operational is diminished. In addition, the aforementioned study in L5178Y lymphoblasts showed decreased drug uptake with calmodulin inhibitors rather than increased uptake (Miller *et al*., 1992).

It has been reported that calmodulin inhibitors can also reduce cellular non-protein thiol content (Shenoy *et al*., 1983). In prior work, glutathione depletion by L-buthionine–sulphoximine created relative increases in growth delay of 37% in TE-671 and 95–113% in TE-671 MR (Rosenberg *et al*., 1989). It is believed that part of the mechanism of resistance in TE-671 MR is due to elevation of glutathione. The effect of calmodulin inhibitors on thiols, however, occurs at much higher drug concentrations than those achievable *in vivo*. It is not clear, then, whether glutathione depletion is playing a role in the modification of drug resistance in this series of experiments. Additional work would be needed to investigate this further.

The mechanism by which flunarizine modifies tumour blood flow is speculated to be either vasodilation or a change in blood viscosity. We recently demonstrated in a rat tumour model that the increase in tumour blood flow observed with flunarizine, given at $1\,mg\,kg^{-1}$ i.v., is accompanied by a significant increase in perivascular oxygenation within the tumour (Dewhirst *et al*., 1992*b*). Furthermore, the increase in blood flow and oxygenation is not attributable to changes in cardiovascular function, tumour feeding vessel diameter, haemoglobin saturation or microvessel haematocrit. By process of elimination, the most likely explanation for the change in blood flow and oxygenation is a drug-induced change in blood viscosity. We subsequently showed that flunarizine reduces blood viscosity *in vitro* in conditions that simulate the tumour microenvironment (hypoxia, lactic acidosis) (Kavanagh *et al*., 1993). In rat red blood cells, the optimal drug concentration to achieve this effect is $5-10\,mg\,l^{-1}$. In contrast, $50\,mg\,l^{-1}$ yields viscosities not unlike those from non-drug-containing control conditions. Parallel studies of cell density and morphology clearly demonstrate that this parabolic dose effect on viscosity is related to achieving an optimal red cell surface area–volume ratio that is most conducive to deformability. High doses lead to spherocytosis (swelling), which reduces deformability. The results of this current study are in agreement with our prior work with this drug in that the optimal dose range for modification of tumour blood flow was $1-5\,mg\,kg^{-1}$, while doses of $60\,mg\,kg^{-1}$ failed to exhibit any rheological change conducive to modification of tumour blood flow (Kavanagh *et al*., 1993). This result is also consistent with prior work by Wood and Hirst (1989), who demonstrated that optimal radiosensitisation of a murine tumour model was achieved at flunarizine doses of $4-5\,mg\,kg^{-1}$ given i.p. Doses in the range of $50\,mg\,kg^{-1}$ did not result in radiosensitisation.

It has been reported previously that calcium channel blockers (verapamil and flunarizine) enhance melphalan retention and increase its activity in tumours (Robinson *et al.*, 1986). These prior results are somewhat difficult to compare with our study because the physiological data were less complete. Direct measurement of cardiovascular function is necessary for interpretation of results, particularly with calcium channel blockers that have more direct chronotropic and inotropic effects than flunarizine, such as verapamil (Godfraind *et al.*, 1986). In the prior studies, measurements of relative blood flow were made with rubidium-86 uptake, which measures fraction of cardiac output rather than tissue perfusion. Therefore, the effects of the calcium channel blockers on tissue blood flow caused by primary effects on cardiac function would not necessarily be determined. Calcium channel antagonists such as nimodipine and diltiazem have been reported to affect $Na^+$, $K^+$-ATPase activity (Godfraind *et al.*, 1986), and $^{86}Rb$ is a potassium analogue. Use of a potassium analogue to monitor changes in tissue blood flow, then, may produce artifactually influenced results in the presence of calcium channel blockers.

Calcium channel blockers and calmodulin inhibitors have been the subject of extensive investigation as chemosensitisers, particularly in regard to modification of drug resistance. The most active work has been related to modification of MDR, although some work has also been done in non-MDR tumours. We chose to study flunarizine in this context because it is both a calcium channel blocker and a calmodulin antagonist (Lugnier *et al.*, 1984). In addition, flunarizine has been reported to have some antiproliferative activity on its own, presumably via the calmodulin-modulated inhibition of DNA synthesis (Sezzi *et al.*, 1984). We chose flunarizine instead of other available calcium channel blockers for several reasons. First, other drugs that have been studied for this type of application, such as verapamil, do not have calmodulin-modulating activity in a dose range achievable *in vivo*. Secondly, flunarizine, unlike similar drugs, could be administered over a wide dose range without appreciable effects on cardiovascular function (Godfraind *et al.*, 1986). Our physiological results verified the previous reports. There were no deaths from flunarizine from either dose, and no increase in frequency of deaths from melphalan or cyclophosphamide toxicity when flunarizine was added. Follow-up times post therapy were greater than 30 days in the TE-671 group, even at the highest melphalan dose administered (18 mg $kg^{-1}$), which is 75% of the $LD_{10}$. Since no deaths were observed in this group, the dose-modifying factor for toxicity must be less than 1.0 0.75 or 1.33. In addition, plasma pharmacokinetics was not altered by the mild drop in blood pressure at the 60 mg $kg^{-1}$ dose of flunarizine, suggesting that renal function was unaltered.

In conclusion, the results of this study demonstrate that flunarizine, given at a high dose (60 mg $kg^{-1}$), can create sensitisation to melphalan cytotoxicity with a dose-modifying factor ranging from 1.5 to 2.3. The enhancement of drug effect is not related to changes in tissue blood flow, but is significantly correlated with decreased drug egress from tumours. Parallel studies of cyclophosphamide showed no enhancement of drug sensitivity, suggesting that the effects of flunarizine are operational on drug uptake or egress mechanisms that are specific for melphalan.

**Acknowledgements**
Editorial assistance was provided by Ann S Tamariz, ELS. This work was supported by NIH Grants NS 20023, CA 56115, CA 11898, CA 40355 and CA 44640 and by ACS Grant CH-403C.

### References

ACKER JC, DEWHIRST MW, HONORE GM, SAMULSKI TV, TUCKER JA AND OLESON JR. (1990). Blood perfusion measurements in human tumours: evaluation of laser Doppler methods. *Int. J. Hyperthermia*, **6**, 287–304.

ADAMS GE, STRATFORD IJ, GODDEN J AND HOWELLS N. (1989). Enhancement of the anti-tumor effect of melphalan in experimental mice by some vaso-active agents. *Int. J. Radiat. Oncol. Biol. Phys.*, **16**, 1137–1139.

BULLARD DE, SCHOLD SC Jr, BIGNER SH AND BIGNER DD. (1981). Growth and chemotherapeutic response in athymic mice of tumors arising from human glioma-derived cell lines. *J. Neuropathol. Exp. Neurol.*, **40**, 410–427.

CHAFOULEAS JG, BOLTON WE AND MEANS AR. (1984). Potentiation of bleomycin lethality by anticalmodulin drugs: a role for calmodulin in DNA repair (abstract). *Science*, **224**, 1346.

DEWHIRST MW, ONG ET, MADWED D, KLITZMAN B, SECOMB T, BRIZEL D, BONAVENTURA J, ROSNER G, KAVANAGH B, EDWARDS J AND GROSS J. (1992*a*). Effects of the calcium channel blocker flunarizine on tumor microvascular hemodynamics and oxygenation. *Radiat. Res.*, **132**, 61–68.

DEWHIRST MW, ONG ET, KLITZMAN B, SECOMB TW, VINUYA RZ, DODGE R, BRIZEL D AND GROSS JF. (1992*b*). Perivascular oxygen tensions in a transplantable mammary tumor growing in a dorsal flap window chamber. *Radiat. Res.*, **130**, 171–182.

FENTON BM AND SUTHERLAND RM. (1992). Effect of flunarizine on micro-regional distributions of intravascular $HbO_2$ saturations in RIF-1 and KHT sarcomas. *Int. J. Radiat. Oncol. Biol. Phys.*, **22**, 447–450.

FRIEDMAN HS, SCHOLD SC Jr. AND BIGNER DD. (1986*a*). Chemotherapy of subcutaneous and intracranial human medulloblastoma xenografts in athymic nude mice. *Cancer Res.*, **46**, 224–228.

FRIEDMAN HS, SKAPEK SX, COLVIN OM, ELION GB, BLUM MR, SAVINA PM, HILTON J, SCHOLD SC, KURTZBERG J AND BIGNER DD. (1986*b*). Melphalan transport, glutathione levels and glutathione-*S*-transferase activity in human medulloblastoma. *Cancer Res.*, **48**, 5397–5402.

FRIEDMAN HS, SCHOLD SC, MAHALEY MS, COLVIN OM, OAKES WJ, VICK NA, BURGER PC, BIGNER SH, BOROWITZ M, HALPERIN EC, DJANG W, FALLETTA JM, DELONG R, GARVIN JH, DEVIVO DC, NORRIS D, GOLEMBE B, WINTER J, BODZINER RA, SIPAHI H AND BIGNER DD. (1989). Phase II treatment of medulloblastoma and pineoblastoma with melphalan: clinical therapy based on experimental models of human medulloblastoma. *J. Clin. Oncol.*, **7**, 904–911.

GODFRAIND T, MILLER R AND WIBO M. (1986). Calcium antagonism and calcium entry blockade. *Pharmacol. Rev.*, **38**, 321–416.

GUPTA V, SINGH SV, AHMAD H, MEDH RD AND AWASTHI YC. (1989). Glutathione and glutathione-*S*-transferases in a human plasma cell line resistant to melphalan. *Biochem. Pharmacol.*, **38**, 1993–2000.

HAIT WN, MORRIS S, LAZO JS, FIGLIN RJ, DURIVAGE HJ, WHITE K AND SCHWARTZ PE. (1989). Phase I trial of combined therapy with bleomycin and the calmodulin antagonist, trifluoperazine. *Cancer Chemother. Pharmacol.*, **23**, 358–362.

HILL RP AND STIRLING D. (1987). Oxygen delivery and tumour response. In *Eighth International Congress of Radiation Research*, Vol. 2, Fielden EM, Fowler JF, Hendry JH and Scott D. (eds) p. 725. Taylor and Francis: London.

HOROWITZ ME, ETCUBANAS E, CHRISTENSEN ML, HOUGHTON JA, GEORGE SL, GREEN AA AND HOUGHTON PJ. (1988). Phase II testing of melphalan in children with newly diagnosed rhabdomyosarcoma: a model for anticancer drug development. *J. Clin. Oncol.*, **6**, 308–314.

KAELIN WG, SHRIVASTAV S AND JIRTLE RL. (1984). Blood flow to primary tumors and lymph node metastases in SMT-2A tumor-bearing rats following intravenous flunarizine. *Cancer Res.*, **44**, 896–899.

KAVANAGH BD, COFFEY BE, NEEDHAM D, HOCHMUTH RM AND DEWHIRST MW. (1993). The effect of flunarizine on the viscosity of human and rat erythrocyte suspensions in conditions of extreme hypoxia and lactic acidosis. *Br. J. Cancer*, **67**, 734–741.

KIKUCHI Y, MIYAUCHI M, KIZAWA I, OOMORI K AND KATO K. (1986). Establishment of a cisplatin-resistant human ovarian cancer cell line. *J. Natl Cancer Inst.*, **77**, 1181–1185.

LAZO JS, CHEN D-L, GALLICCHIO VS AND HAIT WN. (1986). Increased lethality of calmodulin antagonists and bleomycin to human bone marrow and bleomycin-resistant malignant cells. *Cancer Res.*, **46**, 2236–2240.

LEE FYF AND WORKMAN P. (1986). Altered pharmacokinetics in the mechanism of chemosensitization: effects of nitroimidazoles and other chemical modifiers on the pharmacokinetics, antitumor activity and acute toxicity of selected nitrogen mustards. *Cancer Chemother. Pharmacol.*, **17**, 30–37.

LILLEY ER, ELION GB, DEWHIRST MW, SCHOLD SC Jr, BLUM MR, SAVINA PM, LASKOWITZ DT, BIGNER DD AND FRIEDMAN HS. (1991). Therapeutic analysis of melphalan-resistant human rhabdomyosarcoma xenograft TE-671 MR. *Cancer Res.*, **51**, 3906–3909.

LUGNIER C, FOLLENIUS A, GERARD D AND STOCLET JC. (1984). Bepridil and flunarizine as calmodulin inhibitors. *Eur. J. Pharmacol.*, **98**, 157–158.

MCALLISTER RM, ISAACS H, RONGEY R, PEER M, AU W, SOUKUP SW AND GARDNER MB. (1977). Establishment of a human medulloblastoma cell line. *Int. J. Cancer*, **20**, 206–212.

MILLER L, DEFFIE AM AND ROSE R. (1992). Modulation of melphalan uptake in murine L5178Y lymphoblasts *in vitro* by changes in ionic environment. *Biochem. Pharmacol.*, **43**, 1154–1158.

PARSONS PG, CARTER FB, MORRISON L AND MARY R. (1981). Mechanism of melphalan resistance developed *in vitro* in human melanoma cells. *Cancer Res.*, **41**, 1525–1534.

REDWOOD WR AND COLVIN M. (1980). Transport of melphalan by sensitive and resistant L1210 cells. *Cancer Res.*, **40**, 1144–1149.

ROBINSON BA, CLUTTERBUCK RD, MILLAR JL AND McELWAIN TJ. (1986). Effects of verapamil and alcohol on blood flow, melphalan uptake and cytotoxicity, in murine fibrosarcomas and human melanoma xenografts. *Br. J. Cancer*, **53**, 607–614.

ROSE WC, TRADER MW, DYKES DJ, LASTER WR AND SCHABEL FM. (1978). Therapeutic potentiation of nitrosoureas using chlorpromazine and caffeine in the treatment of murine tumors. *Cancer Treat. Rep.*, **62**, 2085–2093.

ROSENBERG MC, COLVIN OM, GRIFFITH OW, BIGNER SH, ELION GB, HORTON JK, LILLEY E, BIGNER DD AND FRIEDMAN HS. (1989). Establishment of a melphalan-resistant rhabdomyosarcoma xenograft with cross-resistance to vincristine and enhanced sensitivity following buthionine sulfoximine-mediated glutathione depletion. *Cancer Res.*, **49**, 6917–6923.

SEZZI ML, ZUPI G, DE LUCA G, MATERAZZI M AND BELLELLI L. (1984). Effects of a calcium-antagonist (flunarizine) on the *in vitro* growth of B16 mouse melanoma cells. *Anticancer Res.*, **4**, 229–234.

SHENOY MA, BIAGLOW JE, VARNES ME AND HETZEL FW. (1983). Inhibition of cultured human tumor cell oxygen utilization by chlorpromazine. *Adv. Exp. Med. Biol.*, **159**, 359–368.

SNEDECOR GW AND COCHRAN WG. (1967). *Statistical Methods*, 6th edn. Iowa State University Press: Ames.

SOMFAI-RELLE S, SUZUKAKE K, VISTICA BP AND VISTICA DT. (1984). Reduction in cellular glutathione by buthionine sulfoximine and sensitization of murine tumor cells resistant to L-phenylalanine mustard. *Biochem. Pharmacol.*, **33**, 485–490.

STEWART DJ AND EVANS WK. (1989). Non-chemotherapeutic agents that potentiate chemotherapy efficacy. *Cancer Treat. Rev.*, **16**, 1–40.

STRATTON MR, DARLING J, PILKINGTON GJ, LANTOS PL, REEVES BR AND COOPER CS. (1989). Characterization of the human cell line TE-671. *Carcinogenesis*, **10**, 899–905.

VAUPEL P AND MENKE H. (1989). Effect of various calcium antagonists on blood flow and red blood cell flux in malignant tumors. *Prog. Appl. Microcirc.*, **14**, 88–92.

WOOD PJ AND HIRST DG. (1989). Calcium antagonists as radiation modifiers: site specificity in relation to tumor response. *Int. J. Radiat. Oncol. Biol. Phys.*, **16**, 1141–1144.